# A Short-Term Incubation with High Glucose Impairs VASP Phosphorylation at Serine 239 in response to the Nitric Oxide/cGMP Pathway in Vascular Smooth Muscle Cells: Role of Oxidative Stress

**DOI:** 10.1155/2014/328959

**Published:** 2014-03-23

**Authors:** Isabella Russo, Michela Viretto, Gabriella Doronzo, Cristina Barale, Luigi Mattiello, Giovanni Anfossi, Mariella Trovati

**Affiliations:** Internal Medicine and Metabolic Disease Unit, Department of Clinical and Biological Sciences, School of Medicine of the Turin University, San Luigi Gonzaga Hospital, Orbassano, 10043 Turin, Italy

## Abstract

A reduction of the nitric oxide (NO) action in vascular smooth muscle cells (VSMC) could play a role in the vascular damage induced by the glycaemic excursions occurring in diabetic patients; in this study, we aimed to clarify whether a short-term incubation of cultured VSMC with high glucose reduces the NO ability to increase cGMP and the cGMP ability to phosphorylate VASP at Ser-239. We observed that a 180 min incubation of rat VSMC with 25 mmol/L glucose does not impair the NO-induced cGMP increase but reduces VASP phosphorylation in response to both NO and cGMP with a mechanism blunted by antioxidants. We further demonstrated that high glucose increases radical oxygen species (ROS) production and that this phenomenon is prevented by the PKC inhibitor chelerythrine and the NADPH oxidase inhibitor apocynin. The following sequence of events is supported by these results: (i) in VSMC high glucose activates PKC; (ii) PKC activates NADPH oxidase; (iii) NADPH oxidase induces oxidative stress; (iv) ROS impair the signalling of cGMP, which is involved in the antiatherogenic actions of NO. Thus, high glucose, via oxidative stress, can reduce the cardiovascular protection conferred by the NO/cGMP pathway via phosphorylation of the cytoskeleton protein VASP in VSMC.

## 1. Introduction 

Among the factors involved in the huge increase of cardiovascular risk occurring in diabetes mellitus, a pivotal role is played by a reduced synthesis and action of nitric oxide (NO) [1], a substance exerting a major role in vascular biology by a wide array of antihypertensive and antiatherogenic properties [2–4]. As it is widely recognized, in diabetes mellitus there is a reduced synthesis of NO by vascular endothelium, mirrored by a reduction of the so-called “endothelial-dependent relaxation,” that is, the “in vivo” vasodilation induced by agents able to stimulate the endothelial synthesis of NO [5, 6]. More controversial is the impairment of the NO action in diabetic patients: for instance, in some studies the so-called “endothelium-independent” relaxation (i.e. the vasodilation induced by exogenous administration of NO donors) is preserved in the presence of an impaired “endothelial-dependent relaxation” [7–9], whereas in other studies both the endothelial and the non-endothelial-dependent relaxation are impaired [10–14].

Therefore, since the endothelial-independent vasodilation mirrors the response of vascular smooth muscle cells (VSMCs) to NO, it has not been completely clarified as yet whether in the presence of diabetes mellitus these cells show a normal or an impaired response to NO, at least as far as vasodilation is concerned.

One of the main actions of NO is to activate the soluble guanylate cyclase (sGC), with the consequent biosynthesis of cyclic guanosine 3′,5′-monophosphate (cGMP), an ubiquitous intracellular second messenger which mediates a large spectrum of biological processes, such as cell contractility, mobility, growth, and apoptosis: the relevance of cGMP signalling in cardiovascular pathophysiology and therapeutics has been exhaustively reviewed [4, 15]. In particular, cGMP deeply influences VSMC contractility, proliferation, and switch from the contractile “differentiated” to the synthetic/secretory “de-differentiated” phenotype [16]. The influence of cGMP on the cardiovascular system is exerted by activating cGMP-dependent protein kinases and phosphatases [15, 17].

The main cGMP-dependent protein kinase in VSMC is PKG type I [15]: the sequential activation of sGC and PKG plays a crucial role in NO action. In particular, downregulation of both enzymes impairs the NO ability to modulate VSMC functions, leading to excessive proliferation, constriction, and secretory activity, as observed in vascular disorders, and ablation of the PKG gene deeply interferes with NO/cGMP-dependent VSMC relaxation both “in vivo” and “in vitro” [18].

One of the PKG-I actions is the phosphorylation, mainly at serine 239, of the vasodilatory-stimulated phosphoprotein (VASP): VASP is a thin filament-actin binding cytoskeletal protein playing a pivotal role in cell adhesion, motility, and migration and—by binding to actin filaments and stress fibers—in cell contraction [19–23]. Thus, VASP phosphorylation at serine 239 is not only a marker of PKG activation but also a mediator of relevant biological actions exerted by the NO/cGMP/PKG pathway, such as modulation of actin polymerization, cell-cell contacts, and relaxation [19–23].

Dysfunction of the cGMP signalling at any level occurs in many cardiovascular diseases, such as arterial and pulmonary hypertension, atherosclerosis, cardiac hypertrophy, vascular remodelling, myocardial ischemia, and heart failure [15]. The dysfunction of the cGMP signalling in diabetes mellitus needs to be further elucidated, as previously mentioned.

Since hyperglycaemia is the main biochemical feature of diabetes mellitus, we aimed to clarify in this study whether high glucose impairs in VSMC the ability of NO to increase the synthesis of cGMP and to activate the downstream cascade of events leading to VASP phosphorylation; furthermore, we aimed to clarify the mechanisms involved in this putative impairment, with a peculiar emphasis for the oxidative stress, which is deeply involved in the pathogenesis of diabetes vascular complications and mediates the vascular damage induced by hyperglycaemia [24, 25].

In particular, we aimed at investigating the role of a short-term VSMC incubation with high glucose: the rationale of our experimental design “in vitro” is the fact that in the last years it has been observed that acute increases of blood glucose concentrations “in vivo,” the so-called “glucose spikes,” mainly occurring after meals, confer a high cardiovascular risk attributed to acute increases of oxidative stress [26].

Interestingly, in a prospective study carried out in our diabetes clinic, we demonstrated that postprandial blood glucose is a stronger predictor of cardiovascular events and death than fasting blood glucose even after the correction for the long-term glucose control marker haemoglobin A1C [27, 28].

In the light of these clinical observations, we decided to evaluate the influence of short-term incubations with high glucose on the NO/cGMP pathway in VSMC and the potential role of oxidative stress.

We also aimed at evaluating the role of protein kinase C (PKC) in the putative glucose-induced, oxidative stress-mediated impairment of the NO-cGMP signalling in VSMC, since one of the main mechanisms linking high glucose and oxidative stress is the glucose-induced activation of PKC, which in turn activates the superoxide anion generating enzyme nicotinamide adenine dinucleotide phosphate (NADPH) oxidase [29].

## 2. Materials and Methods

### 2.1. Research Design

The study has been carried out in cultured rat aortic VSMC.

- To evaluate whether high glucose reduces the NO ability to increase the VASP phosphorylation at serine 239, aortic VSMCs were incubated for 60 min with the NO donor SNP (100 *μ*mol/L) with or without a 120 min preincubation of 25 mmol/L glucose to measure VASP phosphorylation at serine 239: in the following part of the paper, we will indicate this kind of phosphorylation simply as “VASP phosphorylation.”
- To evaluate whether the glucose-induced impairment of the NO ability to phosphorylate VASP is attributable to a reduction of the NO ability to increase cGMP, cGMP concentrations have been measured in aortic VSMC incubated for 60 min with the NO donor SNP (100 *μ*mol/L) with or without a 120 min preincubation with 25 mmol/L glucose.
- To evaluate whether high glucose reduces the cGMP ability to phosphorylate VASP, VASP phosphorylation has been measured in aortic VSMC incubated for 60 min with the cell-permeable cGMP analog 8-Br-cGMP (500 *μ*mol/L) with or without a 120 min preincubation with 25 mmol/L glucose.
- To evaluate the involvement of oxidative stress on the high glucose-induced impairment of the NO- and cGMP-induced VASP phosphorylation, the experiments described at points (a) and (c) were repeated in the presence of a 20 min preincubation with the ROS scavenging enzymes SOD (300 U/mL) + catalase (250 U/mL).
- To evaluate whether high glucose increases radical oxygen species (ROS) production in VSMC and whether this increase is attributable to a PKC-induced activation of NADPH oxidase, ROS concentrations were measured in VSMC with or without a 180 min incubation with 25 mmol/L glucose, in the absence or in the presence of a 20 min preincubation with the PKC inhibitor chelerythrine (2.5 *μ*mol/L) and the NADPH oxidase inhibitor apocynin (10 *μ*mol/L). To evaluate the putative influence on ROS production of two other ROS sources, that is, the mitochondrial electron transport chain complex and xanthine oxidase, experiments with 25 mmol/L glucose were also repeated in the presence of a 20 min preincubation with their specific inhibitors, that is, rotenone (10 µmol/L) and allopurinol (50 µmol/L), respectively. Finally, as a control experiment for the methods employed, experiments with 25 mmol/L glucose were repeated in the presence of a 20 min preincubation with 300 U/mL SOD + 250 U/mL catalase when hydrogen peroxide was measured and 300 U/mL SOD when superoxide anion was measured.
- To evaluate whether high glucose increases PKC alpha-beta phosphorylation, the phosphorylation of PKC *α*/*β*II was measured in VSMC with or without a 180 min incubation with 25 mmol/L glucose.
- To evaluate whether activation of PKC and NADPH oxidase mediates the influence of high glucose on the VASP phosphorylation in response to cGMP, experiments described at point (c) were repeated in the presence of a 20 min preincubation with 2.5 *μ*mol/L chelerythrine and 10 *μ*mol/L apocynin. Experiments have been also repeated in the presence of 10 µmol/L rotenone, to exclude the influence of the mitochondrial electron transport chain complex.

### 2.2. Chemicals

Minimum essential medium (MEM), bovine serum albumin (BSA), sodium nitroprusside (SNP), 8-Br-cGMP, lucigenin, CaCl_2_, MgCl_2_, 4*β*-phorbol 12-myristate 13-acetate (PMA), superoxide dismutase (SOD), catalase, chelerythrine, apocynin, rotenone, and allopurinol were from Sigma-Aldrich (St. Louis, MO, USA). Dihydrodichlorofluorescin diacetate was from Invitrogen Molecular Probes (Paisley, UK). Compounds used for western blots are detailed in the specific paragraphs.

### 2.3. Animals and VSMC Culture

Experiments have been carried out in VSMC derived from aorta of lean Zucker rats isolated and cultured in our laboratory. In particular, male and age-matched rats (*n* = 4), purchased from Charles River Laboratories (Calco, Italy), were fed with standard rodent chow and water ad libitumuntil 14 weeks old and killed with CO_2_ after 12-hour fasting. Aorta was processed for VSMC isolation, culture, and characterization. Cells were characterized by phenotype and checked for the presence of smooth muscle cell *α*-actin and the absence of factor VIII (staining with a fluorescein isothiocyanate-conjugated antibody specific for factor VIII antigen). Cells were cultured using MEM (containing 5 mmol/L glucose) supplemented with 10% fetal calf serum (FCS), 10 mM glutamine, and antibiotics, and buffered with 10 mM N-Tris (hydroxymethyl) methyl-2-aminoethane-sulphonic acid (TES) and 10 mM N-(2-hydroxyethyl) piperazine-N1-(2-ethanesulphonic acid) (HEPES). For the experiments, cells were used at 3th-4th passage and cultured until 70% confluence was achieved. Then, medium with serum was removed and cells were made quiescent by serum starvation and cultured in MEM containing 0.1% BSA. Experiments have been carried out following the National Institutes of Health Guide for the Care and Use of Laboratory Animals 1996 (7th ed.; Washington, DC: National Academy Press, National Research Council Guide) and approved by our Institution.

### 2.4. Intracellular cGMP Measurement

For intracellular cGMP measurementcells were cultured into 6-well plates with medium containing 10% FCS until 70% confluence was achieved; the medium was then removed and replaced overnight with medium containing 0.1% BSA. At the end of the different incubation periods, medium was removed from each well and 300 *μ*L absolute ethanol was added. A complete evaporation of ethanol was obtained under shaking. Then, the precipitated was dissolved in 300 *μ*L acetate buffer and kept at −70°C until the assays. cGMP was measured by RIA kits (Immuno Biological Laboratories, Hamburg, Germany). The sensitivity of assay was less than 0.3 fmol per 0.1 mL, the specificity was 100% for cGMP, 0.0004% for cAMP, and 0.0001% for guanosine monophosphate (GMP), guanosine diphosphate (GDP), adenosine triphosphate (ATP), and guanosine triphosphate (GTP), and intra-assay coefficient of variation was 4.4%. Results were expressed as picomoles cGMP per milligram cell proteins.

### 2.5. Protein Expression and Extent of Protein Phosphorylation by Western Blot

To evaluate the protein expression and the extent of protein phosphorylation by western blot, VSMC extracts (20 *μ*g) were separated by 10% SDS-PAGE and transferred to Immobilon-P Transfer Membranes (Millipore Co, Bedford, MA, USA). Membranes were incubated with the following primary antibodies: rabbit polyclonal anti-total VASP (1 : 15000) and mouse monoclonal anti-VASP phosphorylation at Ser 239 (1 : 1000) (Santa Cruz Biotecnology Inc., CA, USA); rabbit polyclonal anti-PKC (*α*/*β*/*γ*) (1 : 1000) (Upstate, USA) and rabbit polyclonal phospho-PKC *α*/*β*II (Thr 638/641) (1 : 1000) (Cell Signalling, USA). We used as secondary antibodies anti-rabbit (1 : 10000) or anti-mouse (1 : 50000) conjugated to horseradish peroxidase. All the antibodies were diluted in PBS containing 0.1% Tween-20 (Sigma-Aldrich). Blots were scanned and analyzed densitometrically by the image analyzer 1D Image Analysis software (Kodak, Rochester, NY).

### 2.6. Determination of Cellular Reactive Oxygen Species (ROS)

ROS were measured by using the DCF-DA assay, more specific for detection of hydrogen peroxide, and the lucigenin assay, more specific for the detection of superoxide anion.

#### 2.6.1. The DCF-DA Assay

This assay was carried out by using the sensitive fluorescent indicator 2′,7′-dihydrodichlorofluorescin diacetate (DCF-DA), a diacetylated fluorescence probe which diffuses into the cells, where intracellular esterases cleave the acetyl groups, and is oxidized by H_2_O_2_ to the highly fluorescent 2′,7′-dichlorodihydrofluorescein (DCF) [30].

In particular, VSMCs cultured in 96-multiwell plates (1 × 10^5^ mL^−1^) were incubated with MEM with BSA 0.1% containing 5 or 25 mmol/L glucose for 3 h at 37°C. Positive control cells were incubated with 5 mmol/L glucose in the presence of 4*β*-phorbol 12-myristate 13-acetate (PMA) (10 *μ*mol/L) for 1 h, washed, and loaded with DCF-DA. After treatment, the medium was aspirated and cells were washed with PBS containing 1 mM CaCl_2_ and 1 mM MgCl_2 _ (PBS^+^) and incubated in the dark for 60 min at 37°C in the presence of 10 µM of DCF-DA. After that, cells were washed with PBS^+^ and the emitted DCF fluorescence was collected and measured using a plate fluorometer (GloMax-Multi Detection System, Promega Corporation, Madison, WI, USA) fitted with 490 nm excitation and 520 nm emission filters. Each assay was carried out with six replicates and the fluorescence measure for each treatment was the average value of at least three independent experiments. ROS intracellular levels were expressed as fold increase from values with 5 mmol/L glucose.

#### 2.6.2. The Lucigenin Assay

O_2_^−^ levels were measured by lucigenin-enhanced chemiluminescence method based on light emission from reaction between reduced lucigenin and O_2_^−^ as previously described [31]. Briefly, VSMCs, after a 24 h serum starvation, were resuspended at 5 × 10^5^ cells/mL into a luminometer cuvette containing phosphate buffer and maintained at 37°C for 10 min. After a 5 sec dark adaptation, lucigenin (final concentration 25 *μ*mol/L) was added into the cuvette and chemiluminescence was recorded 3 sec after the last injection over a 60 min period at 1 min intervals by the luminescence reader (GloMax-Multi Detection System, Promega Corporation, Madison, WI, USA). Specificity of reaction for O_2_^−^ was demonstrated by preincubating cells with extracellular SOD (300 U/mL). Chemiluminescence activity unit is the relative light unit and O_2_^−^ intracellular levels were expressed as relative light unit per cell.

### 2.7. Statistical Analysis

Data are expressed as means ± standard error of the mean (S.E.M). Statistical analysis was performed by means of analysis of variance (ANOVA) to determine the statistical significance of dose-response effects and by unpaired Student's* t*-test when only two values were compared.

## 3. Results

### 3.1. High Glucose Reduces the SNP-Induced VASP Phosphorylation at Ser 239

**Figure 1:**
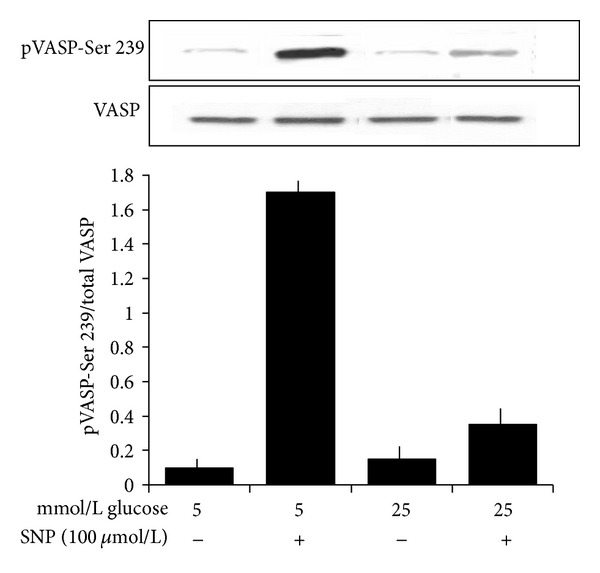
Effects of a 120 min preincubation with 5 and 25 mmol/L glucose on total VASP expression and VASP phosphorylation at Ser 239 in the absence or in the presence of a 60 min incubation with the NO donor SNP (100 *μ*mol/L). Blots are representative of six different experiments. The increase induced by SNP on VASP phosphorylation at both 5 and 25 mmol/L (*P* < 0.0001 and *P* = 0.003, respectively) was lower at 25 than at 5 mmol/L (*P* < 0.0001). SNP did not modify total VASP expression neither in the absence nor in the presence of high glucose.

As shown in Figure 1, (i) high glucose did not modify VASP expression and phosphorylation in the absence of SNP; (ii) SNP did not modify total VASP expression neither in the absence nor in the presence of high glucose; (iii) SNP induced a significant VASP phosphorylation in the presence of both 5 mmol/L (*n* = 6, *P* < 0.0001) and 25 mmol/L glucose (*n* = 6, *P* = 0.003), the percent values on baseline being 602.4 ± 17% and 165.7 ± 10.9%, respectively. In the presence of 25 mmol/L glucose, values were significant lower than in the presence of 5 mmol/L glucose (*P* < 0.0001).

### 3.2. High Glucose Does Not Modify the SNP-Induced Increase of cGMP

**Figure 2:**
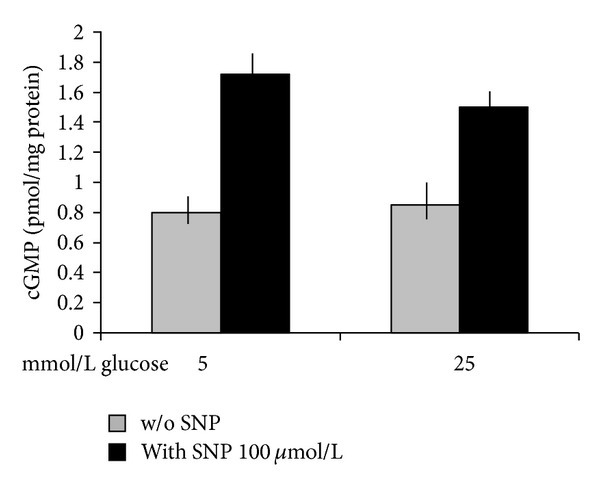
Effects of a 120 min preincubation with 5 and 25 mmol/L glucose on cGMP production in the absence or in the presence of a 60 min incubation with the NO donor SNP (100 *μ*mol/L). The increase induced by SNP at both 5 and 25 mmol/L (*n* = 6, *P* < 0.0001 for both) was similar for both glucose concentrations (*P* = ns).

As shown in Figure 2, SNP induced a significant increase of cGMP concentrations in the presence of both 5 mmol/L glucose (*n* = 6, *P* < 0.0001) and 25 mmol/L glucose (*n* = 6, *P* < 0.0001), without significant differences between the two glucose concentrations.

### 3.3. High Glucose Reduces the cGMP-Induced VASP Phosphorylation at Ser 239

**Figure 3:**
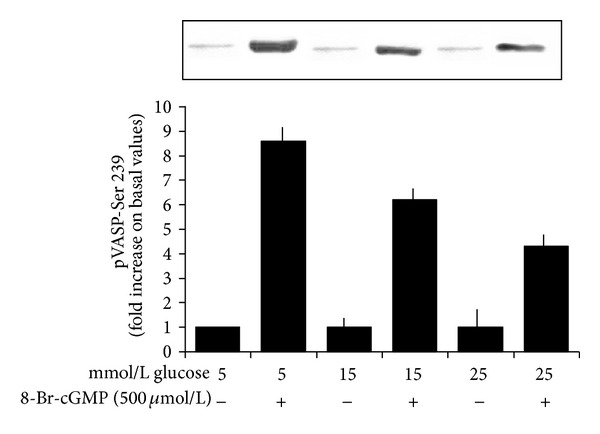
Effects of a 120 min preincubation with 5, 15, and 25 mmol/L glucose on VASP phosphorylation at Ser 239 in the absence or in the presence of a 60 min incubation with the cell-permeable cGMP analog 8-Br-cGMP (500 *μ*mol/L). Blots are representative of six different experiments. 8-Br-cGMP induced a significant VASP phosphorylation at the three glucose concentrations (*P* < 0.0001 for each of them). When values are expressed as percent of values at glucose 5 mmol/L, the extent of VASP phosphorylation induced by 8-Br-cGMP dose dependently decreased (ANOVA: *P* < 0.0001).

As shown in Figure 3, 8-Br-cGMP induced a significant VASP phosphorylation in the presence of 5 mmol/L (*n* = 6, *P* < 0.0001), 15 mmol/L (*n* = 6, *P* < 0.0001), and 25 mmol/L glucose (*n* = 6, *P* < 0.0001). When values are expressed as percent of baseline values at glucose 5 mmol/L, the extent of VASP phosphorylation induced by 8-Br-cGMP dose dependently decreased (ANOVA: *P* < 0.0001).

### 3.4. The Antioxidant Mixture SOD + Catalase Prevents the Inhibitory Effects Exerted by High Glucose on the VASP Phosphorylation Induced by SNP and by 8-Br-cGMP

**Figure 4:**
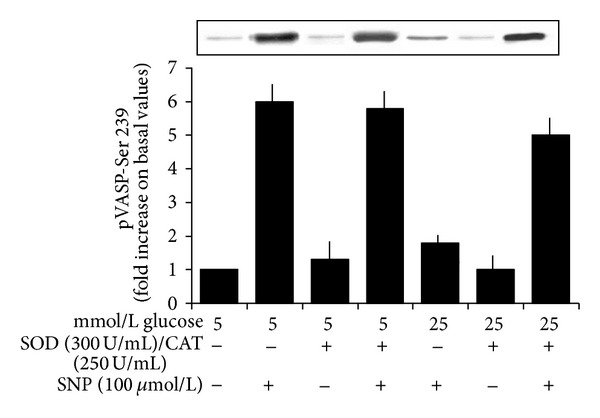
Effects of a 20 min preincubation with the antioxidant mixture SOD (300 U/mL) + catalase (250 U/mL) on the SNP-induced VASP phosphorylation at Ser 239 in the presence of 5 and 25 mmol/L glucose. Blots are representative of six different experiments. SOD and catalase did not modify the significant increase of VASP phosphorylation induced by SNP in the presence of 5 mmol/L glucose but significantly increased the extent of the SNP-induced VASP phosphorylation in the presence of glucose 25 mmol/L (*P* < 0.0001).

**Figure 5:**
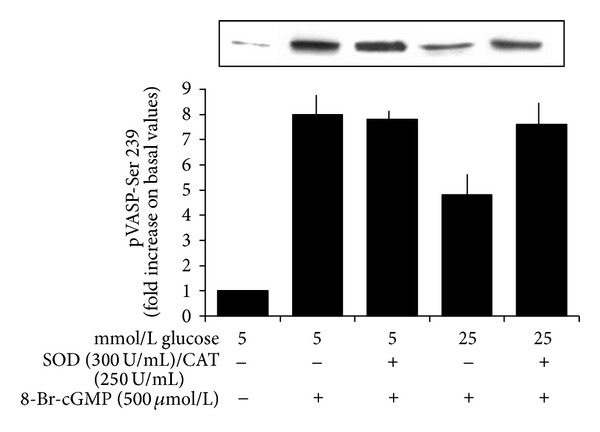
Effects of a 20 min preincubation with the antioxidant mixture SOD (300 U/mL) + catalase (250 U/mL) on the 8-Br-cGMP-induced VASP phosphorylation at Ser 239 in the presence of 5 and 25 mmol/L glucose. Blots are representative of six different experiments. SOD and catalase did not modify the significant increase of VASP phosphorylation induced by 8-Br-cGMP in the presence of 5 mmol/L glucose but significantly increased the extent of 8-Br-cGMP-induced VASP phosphorylation in the presence of glucose 25 mmol/L (*P* < 0.0001).

As shown in Figures 4 and 5, the antioxidant mixture SOD + catalase did not modify the extent of VASP phosphorylation induced by SNP or 8-Br-cGMP in the presence of 5 mmol/L glucose but restored the inhibitory effects induced by 25 mmol/L glucose (*n* = 6, *P* < 0.0001 for both).

### 3.5. The High Glucose-Induced Increase of ROS Production Is Prevented by PKC and NADPH Oxidase Inhibitors

**Figure 6:**
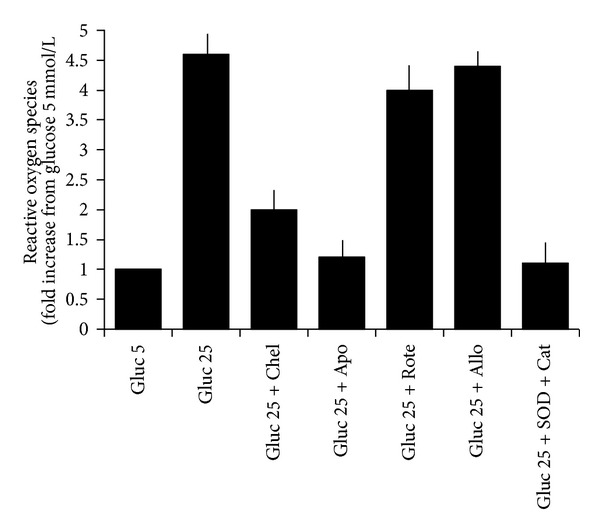
Effects of a 180 min incubation with 5 and 25 mmol/L glucose on ROS concentrations in the absence or in the presence of a 20 min preincubation with the PKC inhibitor chelerythrine (2.5 *μ*mol/L), the NADPH-oxidase inhibitor apocynin (10 *μ*mol/L), the mitochondrial electron transport chain complex inhibitor rotenone (10 µmol/L), the xanthine oxidase inhibitor allopurinol (50 µmol/L), and a mixture of SOD and catalase (300 U/mL/250 U/mL) (*n* = 6). ROS values in the presence of 25 mmol/L glucose were significantly higher than in the presence of 5 mmol/L glucose (*n* = 6, *P* < 0.0001). SOD + catalase, chelerythrine, and apocynin blunted the effects of glucose 25 mmol/L (*P* < 0.0001 for each), whereas rotenone and allopurinol did not modify the high glucose effects (*P* = ns).

As shown in Figure 6, a 180 min incubation with 25 mmol/L glucose increased ROS production, measured by the DCF-DA assay specific for H_2_O_2_ (*n* = 6, *P* < 0.0001). This increase was inhibited by preincubation with the PKC inhibitor chelerythrine (2.5 *μ*mol/L) (*n* = 6, *P* < 0.0001), the NADPH-oxidase inhibitor apocynin (10 *μ*mol/L) (*n* = 6, *P* < 0.0001), and, as expected, by SOD (300 U/mL) + catalase (250 U/mL) (*n* = 6, *P* < 0.0001). With the three inhibitors, ROS values were similar to those measured in the presence of 5 mmol/L glucose (*P* = ns). ROS production was unaffected by incubation with 10 µmol/L rotenone and 50 µmol/L allopurinol (*P* = ns versus 25 mmol/L glucose for both).

Similar results have been obtained by the lucigenin assay, specific for O_2_^−^. In particular, when values are expressed as percent of baseline values at 5 mmol/L glucose, in the presence of 180 min incubation with 25 mmol/L glucose the O_2_^−^ production was 144.7 ± 22.5% (*n* = 6, *P* < 0.0001): this increase was completely inhibited by preincubation with the PKC inhibitor chelerythrine (2.5 *μ*mol/L) (*n* = 6, *P* < 0.0001), the NADPH-oxidase inhibitor apocynin (10 *μ*mol/L) (*n* = 6, *P* < 0.0001), and, as expected, by SOD (300 U/mL) (*n* = 6, *P* < 0.0001). With the three inhibitors, O_2_^−^ values were similar to those measured in the presence of 5 mmol/L glucose (*P* = ns).

### 3.6. High Glucose Increases PKC Alpha/Beta Phosphorylation

**Figure 7:**
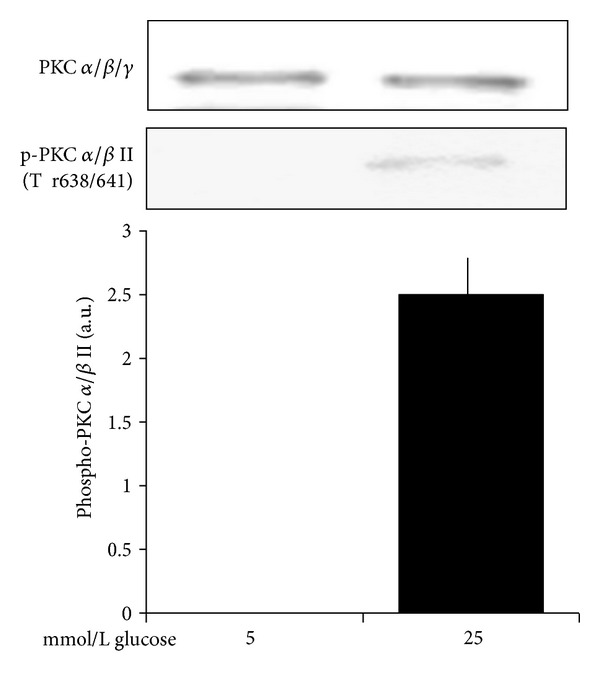
Effects of a 180 min incubation with 5 and 25 mmol/L glucose on nonphosphorylated PKC *α*/*β*/*γ* isoforms and on PKC *α*/*β*II phosphorylated at Thr 638/641. Blots are representative of four different experiments. Glucose 25 mmol/L did not modify the expression of nonphosphorylated PKC *α*/*β*/*γ* isoforms and increased PKC *α*/*β*II phosphorylation (*P* < 0.0001).

As shown in Figure 7, 25 mmol/L glucose induces a PKC *α*/*β*II activating phosphorylation without modifying protein expression. In particular, a 180 min incubation with 25 mmol/L glucose in comparison to 5 mmol/L glucose (i) did not modify the expression of total PKC *α*/*β*/*γ* (*n* = 4, *P* = ns); (ii) increased the phosphorylation of PKC *α*/*β*II at Thr 638/641 (*n* = 4, *P* < 0.0001).

### 3.7. In the Presence of High Glucose, the cGMP-Induced VASP Phosphorylation Is Increased by PKC and NADPH Oxidase Inhibitors

**Figure 8:**
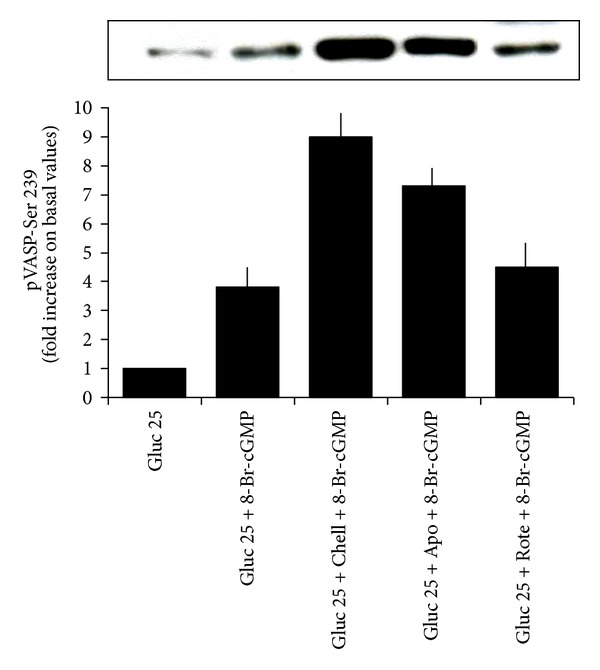
Effects of a 20 min preincubation with the PKC inhibitor chelerythrine (2.5 *μ*mol/L), the NADPH-oxidase inhibitor apocynin (10 *μ*mol/L), and the mitochondrial electron transport chain complex inhibitor rotenone (10 µmol/L), on the 8-Br-cGMP-induced VASP phosphorylation at Ser 239 in the presence of 25 mmol/L glucose. Blots are representative of four different experiments. Chelerythrine and apocynin significantly increased the extent of 8-Br-cGMP-induced VASP phosphorylation in the presence of glucose 25 mmol/L (*P* < 0.0001 for both), which was not modified by rotenone (*P* = ns).

As shown in Figure 8, the VASP phosphorylation induced by 8-Br-cGMP in the presence of 25 mmol/L glucose was significantly enhanced by both 2.5 *μ*mol/L chelerythrine and 10 *μ*mol/L apocynin (*n* = 4, *P* < 0.0001 for both) and was unaffected by 10 µmol/L rotenone.

## 4. Discussion

This study shows that, in cultured rat aortic VSMC, a short-term incubation with high glucose impairs the NO-induced VASP phosphorylation at serine 239 and that this effect is not due to a reduced cGMP synthesis but due to a reduced cGMP action and involves oxidative stress. The proposed mechanism is the following sequence of events: (i) high glucose activates PKC; (ii) PKC activates NADPH oxidase; (iii) NADPH oxidase increases the production of superoxide anion, and, consequently, of hydrogen peroxide; (iv) ROS impair the cGMP ability to phosphorylate VASP at serine 239.

As far as we know, this study provides the first evidence of the high glucose ability to reduce the NO/cGMP-induced VASP phosphorylation in cultured VSMC: a previous study, carried out with a long-term incubation with high glucose, demonstrated a similar inhibition in cultured human lung microvascular endothelial cells [32]. A reduced VASP phosphorylation was also observed in endothelial progenitor cells derived from two diabetic patients and therefore exposed to high glucose “in vivo” [32].

Our study also shows that oxidative stress plays a pivotal role in the high glucose-induced impairment of the NO/cGMP signalling in VSMC, since this impairment is completely prevented by the antioxidant mixture SOD + catalase.

As it is well known, during the physiological cellular metabolism oxygen undergoes a cascade of reductions, leading to the sequential production of superoxide anion, which is dismutated by superoxide dismutases (SOD) to hydrogen peroxide, which is catalyzed to H_2_O by catalase; superoxide anion and hydrogen peroxide belong to the class of the “Reactive Oxygen Species” (ROS); excessive increases of ROS lead to the so-called “oxidative stress,” a common phenomenon in many vascular diseases, such as diabetes mellitus, arterial hypertension, hypercholesterolemia, and heart failure, as reviewed [33–35].

In our study, SOD and catalase completely prevented the inhibiting influence exerted by high glucose on the VASP phosphorylation induced by the NO/cGMP signalling, underlining the role of oxidative stress in this phenomenon.

Our working hypothesis was that high glucose increases in a short-term oxidative stress by a PKC-induced activation of NADPH oxidase; this hypothesis has been confirmed by the experiments carried out by measuring ROS concentrations, in which both PKC and NADPH oxidase inhibition impaired the stimulating effect of high glucose, whereas inhibitors of other potential ROS sources, such as mitochondrial respiratory chain complex and xanthine oxidase, did not modify the high glucose effects. Interestingly, PKC and NADPH oxidase inhibitors were also able to restore the extent of the cGMP-induced VASP phosphorylation impaired by high glucose. As it is well known, NADPH oxidase is the major source of ROS in VSMC [36]. We recently demonstrated that also oleic acid increases oxidative stress in VSMC by a mechanism involving both PKC and NADPH oxidase [37]. Previous observations carried out in our laboratory demonstrated that a 24 h incubation of rat VSMC with hydrogen peroxide reduces the cGMP-induced VASP phosphorylation, indicating a peculiar role of oxidative stress in the impairment of cGMP signalling [31].

In rat aortic VSMC, it has been demonstrated that a long-term (24–48 h) incubation with high glucose in the absence of exposure to NO donors or cGMP reduces the constitutive PKG-1 synthesis and, consequently, the PKG-induced VASP phosphorylation; also in this case the phenomenon is prevented by NADPH oxidase and PKC inhibitors [38]. The same authors demonstrated that a 3 h incubation with high glucose failed to modify PKG-1 expression [38]; thus, the results we obtained in the present study cannot be attributed to a reduced synthesis of PKG.

Independently of high glucose, a ROS-mediated reduction of PKG activity without any change in PKG expression has been observed in cultured VSMC from ovine fetal intrapulmonary veins exposed for 30 min to hypoxia and attributed to posttranslational, ROS-induced PKG nitration in tyrosine residues [39]; interestingly, the ROS-mediated downregulation of PKG activity was more evident in the presence of cGMP, suggesting that one or more residues within the cGMP-binding region of PKG are susceptible to ROS-induced posttranslational modifications [39]. In agreement with this observation, in our experimental conditions the extent of VASP phosphorylation—a reliable marker of PKG activity in vascular tissue [19]—was reduced by high glucose via oxidative stress only in the presence of NO or cGMP.

In conclusion, our study originally demonstrates the ability of high glucose to influence the NO/cGMP/PKG/VASP pathway in isolated, cultured VSMC by a mechanism involving the increase of oxidative stress mediated by a PKC-induced enhancement of the NADPH oxidase activity. It therefore provides some new information to further explain the interesting results of previous investigations carried out in aortas from rats sacrificed weeks after a streptozotocin injection, representing a classical animal model of “in vivo” hyperglycaemia [40, 41]. Obviously, when hyperglycaemia occurs “in vivo,” high glucose affects many different tissues with the occurrence of the well-known intercellular interplay mediated by the release of different molecules, a phenomenon prevented by the “in vitro” incubation with high glucose of isolated cells. In any case, in these “in vivo” studies it has been demonstrated that streptozotocin-induced diabetes causes an impairment of endothelium-dependent [40, 41] and endothelium-independent vasodilation [41] and increases NADPH oxidase activity and expression and superoxide production in aorta [40, 41], the last phenomenon being prevented by the incubation of aortic rings with PKC inhibitors and by their “in vivo” administration [40]. Interestingly, “in vivo” administration of a PKC inhibitor markedly decreased superoxide anion production both in the endothelial and in the media layers of the aorta, indicating the occurrence of a PKC-mediated increase of oxidative stress in VSMC, the main component of the media [40].

Furthermore, acetylcholine induced an increase of VASP phosphorylation at serine 239 in aortic tissue of control rats, but not in that of rats with streptozotocin-induced diabetes; owing to the experimental design, this phenomenon has been attributed to the marked reduction of the acetylcholine-induced NO production in the vascular endothelium [41].

Our study, carried out in isolated cultured VSMC, adds a further piece of information on the mechanisms of glucose-induced impairment of VASP phosphorylation in vascular tissues, since it clarifies that in VSMC this impairment is due to a defect of the cGMP signalling and therefore occurs also independently of the reduced NO synthesis and bioavailability caused by the glucose effects on vascular endothelium.

Finally, our present study provides the first demonstration of the ability of high glucose to rapidly reduce the NO/cGMP signalling in VSMC; this fact could be relevant to explain one of the possible mechanisms by which the so-called “glucose spikes” occurring “in vivo” in diabetic patients negatively influence vascular function [26].

## 5. Conclusions

In conclusion, in cultured aortic VSMC a short-term incubation with high glucose reduces the ability of both NO and cGMP to phosphorylate VASP at Ser 239 with a mechanism mediated by oxidative stress. As described in the Introduction, VASP phosphorylation is deeply involved in many antihypertensive and antiatherogenic biological actions exerted by the NO/cGMP/PKG pathway, such as modulation of cell adhesion, motility, migration, and contraction. Since the impairment of the NO pathway plays a pivotal role in the pathogenesis of the atherothrombotic vascular complications of diabetes, our results, by identifying a potential mechanism involved in the reduced NO action in VSMC, could have a clinical relevance. In particular, the results of our study can clarify another mechanism of the harmful vascular consequences of the so-called “glucose spikes” occurring “in vivo” in diabetic patients, which have been attributed to acute increases of oxidative stress, indicating an involvement of VSMC beyond the previously described involvement of vascular endothelium.
